# Efficient propagation of suspended HL-60 cells in a disposable bioreactor supporting wave-induced agitation at various Reynolds number

**DOI:** 10.1007/s00449-020-02386-6

**Published:** 2020-06-09

**Authors:** Kamil Wierzchowski, Iwona Grabowska, Maciej Pilarek

**Affiliations:** 1 Faculty of Chemical and Process Engineering, Warsaw University of Technology, Waryńskiego 1, 00-645 Warsaw, Poland; 2 Faculty of Biology, University of Warsaw, Miecznikowa 1, 02-096 Warsaw, Poland

**Keywords:** Disposable (single-use) bioreactor, Wave-type agitation, Re number, *k*_L_*a* coefficient, Human hematopoietic HL-60 cells, Nonadherent cell propagation

## Abstract

Growth of human nonadherent HL-60 cell cultures performed in disposable bioreactor under various hydrodynamic conditions of 2-D wave-assisted agitation has been compared and discussed. Influence of Reynolds number for liquid (Re_L_) and the *k*_L_*a* coefficient, as key parameters characterized the bioprocessing of HL-60 cells in *ReadyToProcess* WAVE^TM^ 25 system, on reached values of the apparent maximal specific growth rate (*μ*_max_) and the specific yield of biomass (Y^*^_X/S_) has been identified. The values of Re_L_ (i.e., 510–10,208), as well as *k*_L_*a* coefficient (i.e., 2.83–13.55 h^−1^), have been estimated for the cultures subjected to wave-induced mixing, based on simplified dimensionless correlation for various presents of WAVE 25 system. The highest values of apparent *μ*_max_ = 0.038 h^−1^ and Y^*^_X/S_ = 25.64 × 10^8^ cells g_glc_^−1^ have been noted for cultures independently performed at wave-induced agitation characterized by Re_L_ equaled to 5104 and 510, respectively. The presented results have high applicability potential in scale-up of bioprocesses focused on nonadherent animal cells, or in the case of any application of disposable bioreactors presenting similitude.

## Introduction

The modern biopharmaceutical industry produces and commercially offers a range of animal-based bioproducts obtained through in vitro bioprocessing of mammalian and human cells, which can roughly be divided into two basic morphological categories: anchorage-dependent (adherent) cells and free-floating (nonadherent) ones. As fragile biomass, animal cells require gentle flow of liquid phase in culture vessel, which is characterized by low values of Reynolds number for liquid phase (Re_L_), as well as low levels of hydrodynamic shear stress [1, 2]. Feasibility of gently mixing in small-scale wave-type agitated single-use (disposable) bioreactors establishes applicability of such systems for in vitro scale-up of fragile biomass of both nonadherent and adherent animal cells [3–5].

In the case of nonadherent cells, typically applied static (i.e., unagitated) cultures do not provide sufficient conditions for efficient propagation of suspended cells, in general. Instead of commonly applied rotating or tumbling stirrers used to induce fluid flow in classical bioreactor systems, continuously oscillating devices can be utilized for a gentle obtaining of homogeneous conditions in the systems for in vitro culture of the fragile animal cells. In such approaches, the agitation is achieved by 2-D horizontal oscillations of the disposable culture bag fixed in a rocker unit [6]. The continuous rocking motion of the culture vessel induces waves in the two-phase (gas–liquid) culture system composed of CO_2_-enriched air and suitable culture medium closed inside the culture bag. Generated waves cause agitation of large volumes of medium, and facilitate dispersion of culture microenvironment components, i.e., gases, nutrients, extracellularly secreted waste bioproducts [7, 8], which finally enhances homogeneity of the culture environment inside the disposable culture bag. Thus, efficient in vitro propagation of animal cells can be performed at the sufficient level of aeration and satisfactory values of the *k*_L_*a* coefficient reached at low level of Re_L_ number characterizing wavy flow of culture medium in such single-use bioreactor platforms [9, 10].

Generally, nonadherent hematopoietic cells are recognized as relatively more sensitive on hydrodynamics effects in cultures than some typical industrially applied cell lines exhibiting anchorage dependency, which are a bit more robust to shear forces, especially if they are cultured on suitable microcarriers [11, 12]. However, hematopoietic cells are an acknowledged ex vivo source of bioproducts with many potential applications in bone marrow transplantation, immunotherapy, gene therapy, as well as the source of blood-derived products [13]. A HL-60 cell line, i.e., human acute promyelocytic leukemia cells, has been frequently applied as model of hematopoietic cells, and it was used in studies focused on in vitro blood cell formation [14], their physiology [15] and differentiation [16, 17], as well as to studying the cytotoxicity effects of potential anti-leukemic drugs [18], or even in lab-on-a-chip applications [19]. Thus, an enhancement of HL-60 cells’ propagation efficiency is a current research issue highly expected by representatives of a range of application niches, which experimentally involve such type of cells.

The basic aim of the study was to recognize in detail the outcomes of nonadherent HL-60 cell cultures performed under various hydrodynamic conditions of wave-type agitation in a disposable bioreactor. The influence of Re_L_ and the *k*_L_*a* coefficient, as parameters characterized bioprocessing of HL-60 cells performed in *ReadyToProcess* WAVE™ 25 system, on reached values of the specific yield of biomass (Y^*^_X/S_), as well as the apparent maximal specific growth rate (*μ*_max_), has been determined and discussed. Activity of extracellularly leakage lactate dehydrogenase (*a*_LDH_), specific glucose consumption rate per cell (r^*^_glc/cell_), as well as level of intracellular dehydrogenases activity (*a*_m_) have been also discussed to comprehensively present complete set of data and to make them ready to interpret.

In our opinion, such detailed and specific discussion, comprehensively grounded by complete set of reference data, is highly expected by specialists in the field of scale-up bioprocesses with nonadherent hematopoietic cells, or for any other bioengineers/biotechnologists focused on propagation of animal/mammalian cells in other disposable bioreactor systems or spinner flasks, as well as in the case of any purpose for scale-up of laboratory-scale cultures.

## Materials and methods

### Disposable bioreactor system

**Fig. 1:**
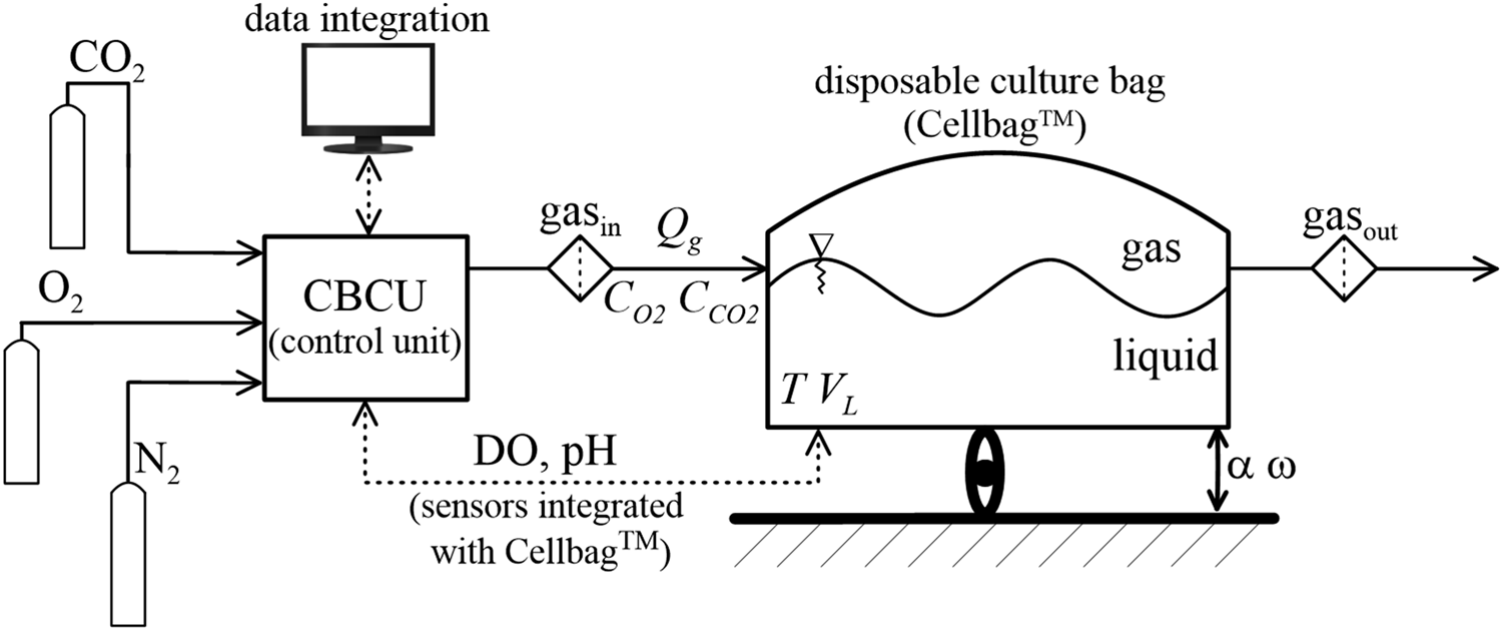
Schematic diagram of WAVE 25

*ReadyToProcess* WAVE™25 system (WAVE 25, GE Healthcare Bio-Sciences, US) has been applied to perform in vitro culture of nonadherent HL-60 cells under conditions of continuously wave-type agitation. The schematic diagram of WAVE 25 setup has been presented in Fig. 1. WAVE 25 was equipped with disposable pre-sterilized polymer-based Cellbag™ (Cellbag, GE Healthcare, US) as the flexible culture vessel with 2.0 L of total volume, which can be filled with 0.1–1.0 L (as recommended working volume for liquid phase) with culture medium/broth. More technical details about the used configuration of WAVE 25 bioreactor are available in some previous reports, e.g. [10].

Cellbag was continuously rocked on a rocking tray of WAVE 25 and wave-induced agitation has been defined by two parameters of two-dimensional (2-D) rocking motion: oscillation angle (*α*) and oscillation frequency (*ω*). The control unit (CBCU) allowed to integrate crucial data of the bioprocess, such as pH level and DO (both signals were independently transmitted via two optical fibers connected with two miniaturized optical sensors of pH and DO built-in inside the bottom of Cellbag), the total flow rate (*Q*_G_) of inlet gas mixture (gas_in_) through Cellbag (i.e., over waving liquid phase), as well as concentrations of O_2_ (*C*_O2_) and CO_2_ (*C*_CO2_) in gas_in_.

### HL-60 cells and culture medium

HL-60 is a referenced continuous line of nonadherent human promyelocytic leukemia white blood cells originally isolated from blood of a woman (36 y.o.) in 1979. HL-60 line is standardly applied in laboratory researches on in vitro blood cell formation and blood physiology. The cells applied in the study were referentially certified as HL-60 cell line supplied by ATCC (US).

In all experiments, HL-60 cells were maintained in the culture medium composed as a mixture of 89% of Roswell Park Memorial Institute 1640 medium (RPMI) containing 2.0 g L^−1^ of glucose, 10% of inactivated fetal bovine serum (FBS), and 1% of commercial antibiotic/antimycotic mixture (PenStrep). All applied liquid media and additives were supplied by Thermo Fisher Scientific (Gibco™ culture media, US) as certified components approved for in vitro cultures of animal cell.

### Maintaining of HL-60 cells

In the case of experimental batch cultures subjected to wave-induced agitation, HL-60 cells have been maintained in 2-L Cellbag containing 300 mL of culture medium and incubated at the operating parameters of WAVE 25 presented in Table 1. In each case, the disposable culture system was stabilized at 37 °C, as well as DO value was equaled to 100% saturation of the liquid phase with O_2_ from sterile gas_in_ composed as 74% N_2_ + 21% O_2_ + 5% CO_2_ mixture of pure gases, and dosed with *Q*_*G*_, prior to inoculation (*X*_0_ = 1 × 10^5^ cells mL^−1^) as the starting point of each culture. All cultures established in WAVE 25 system were triplicated, and all the cultures were maintained for 7 days, with daily harvesting of samples.

**Table 1:** Operating parameters of WAVE 25 bioreactor applied as conditions of HL-60 cell cultures performed under wave-induced agitation

Operating parameters	Value	Unit
Variables		
* α*	2, 6, 12	°
* ω*	2, 20, 40	min^−1^
Constants		
* Q*_G_	0.5	L min^−1^
* V*_L_	0.3	L
* C*_O2_	21	%
* C*_CO2_	5	%
* T*	37	°C

### Analytical methods

Analytical methods performed with samples harvested from independent cultures allow to separately characterize biomass and culture medium. For following days of each culture, values of *X*, *Z*, *a*_m_ and r^*^_glc/cell_ were determined as quantitative characteristics of biomass, as well as DO and pH levels and *a*_LDH_, were determined as quantitative characteristics of culture medium.

*X* and *Z *were determined based on results of HL-60 cell staining with 0.4% trypan blue aqueous solution (Thermo Fischer Scientific, US). After 3-min incubation at 37 °C of the mixed equimolar samples of the cells suspension and the dye, live (i.e., unstained) and dead (i.e., blue-stained) cells were manually counted in the Bürker-Türk haemocytometer (Brand, DE) under Eclipse TS100 reverse microscope (Nikon, JP). Next, the values of *X* and *Z* were finally calculated from the following equations:

1 $$X = \frac{x}{k} \cdot d \cdot 5 \cdot 10^{5} \; [{\text{cells mL}}^{{ - {1}}} ],$$

where *x* is the total number of cells (i.e., summarized both stained and unstained ones) counted in the grid of the hemocytometer, *k* is the number of grid squares with cells and *d* is the dilution of the sample containing cells;

2 $$Z = \frac{z}{x} \cdot 100\% ,$$

where *z* is the number of living (i.e., unstained) cells.

*a*_m_ has been recognized as the parameter quantitatively characterizing mitochondrial activity of HL-60 cells by the estimation of intracellular oxidoreductase activity level, according to resazurin-based PrestoBlue™ assay (PrestoBlue, Thermo Fischer Scientific, US). 0.1 mL of PrestoBlue reagent was mixed with 0.9 mL sample of cells suspension (in the case of test samples) or with 0.9 mL of pure culture medium without cells (in the case of reference samples). Next, all samples were incubated for 10 min at 37°, and finally specific absorbance was measured using GENESYS 20 UV–VIS spectrophotometer (Thermo Fisher Scientific, US) at 570 nm vs. referenced 600 nm. Finally, values of *a*_m_ were calculated as follows:

3 $$a_{{\text{m}}} = 37.04 \cdot A_{{\text{W}}} \;\left[ {\mu {\text{kat L}}^{{ - {1}}} } \right],$$

where *A*_W_ is the specific absorbance of the sample.

r^*^_glc/cell_ has been estimated based on daily monitored glucose level in culture medium by BioMaxima-glucose enzymatic assay (BioMaxima, PL). 1.0 mL of BioMaxima reagent was mixed with 20 μL of filtered (*φ* = 0.2 µm) culture medium harvested daily from the cultures (in the case of test sample) or with 20 μL of double-distilled water (in the case of blank sample). Next, all samples were incubated for 20 min at room temperature prior to measurements of absorbance in GENESYS 20 UV–VIS spectrophotometer (Thermo Fisher Scientific, US) at 500 nm. Finally, values of r^*^_glc/cell_ were calculated according to following equation:

4 $$r_{{\text{glc/cell}}}^{*} = \frac{{\Delta C_{{{\text{glc}}}} }}{\Delta t \cdot X}\; \left[ {{\text{g h}}^{{ - {1}}} {\text{cell}}^{{ - {1}}} } \right],$$

where Δ*C*_glc_ is the daily change in glucose concentration in the culture medium and Δ*t* is the time interval between two consecutive measurements of Δ*C*_glc_.

*a*_LDH_ has been determined based on daily monitored activity level of intracellular lactate dehydrogenase (LDH), which leaked only from damaged (i.e., interpreted as dying) HL-60 cells into culture medium. The colorimetric kinetic determination according to theprocedure of BioMaxima-LDH enzymatic assay (BioMaxima, PL) has been applied in our study. 1.0 mL of Biomaxima-LDH reagent was added to 10 μL of filtered (*φ* = 0.2 µm) culture medium harvested daily from cultures (in the case of test samples) or mixed with 10 μL of double-distilled water (in the case of blank sample). Absorbances of such reaction mixtures were spectrophotometrically monitored in 1-min intervals in GENESYS 20 UV–VIS spectrophotometer (Thermo Fisher Scientific, US) at 340 nm. Finally, values of *a*_LDH_ were finally estimated based on the following equation:

5 $$a_{{{\text{LDH}}}} = 267.2 \cdot \Delta A\; [\mu {\text{kat L}}^{{ - {1}}} ],$$

where Δ*A* is the absorbance change *per* minute.

DO as well as pH level have been automatically measured by miniaturized spot-like sensors built-in inside the bottom of Cellbag. The correctness of both values were verified and certified by the manufacturer of the culture bag according to setting the blank data for both sensors, which have been printed individually on each applied Cellbag, into operating system of WAVE 25.

### Mathematical methods

Values of Re_L_, as well as *k*_L_*a*, as key parameters quantitatively characterizing hydrodynamic conditions of bioprocesses performed in WAVE 25 bioreactor operated at various sets of operational parameters (*α* and *ω*) have been calculated based on two correlations originally introduced and published previously [10]. Based on values of *α* and *ω*, the values of Re_L_ can be calculated as follows [10]:

6 $${\text{Re}}_{{\text{L}}} = \frac{{\omega L^{2} }}{{v_{{\text{L}}} }}\sin \alpha ,$$

where *L* is length of culture bag and *v*_L_ is liquid phase kinematic viscosity.

While the values of *k*_L_*a* for the WAVE 25 bioreactor can be predicted based on the following dimensionless correlation [10]:

7 $$k_{{\text{L}}} a = 13.423 {\text{Re}}_{{\text{L}}}^{0.522} {\text{Re}}_{{\text{G}}}^{0.664} {\text{Sc}}^{0.572} \frac{{D_{{\text{L}}} }}{{L^{2} }}\; \left[ {{\text{s}}^{{ - {1}}} } \right],$$

where Re_G_ is Reynolds number for gas phase, Sc is Schmidt number for liquid phase, and *D*_L_ is coefficient of oxygen diffusivity in liquid phase.

**Fig. 2:**
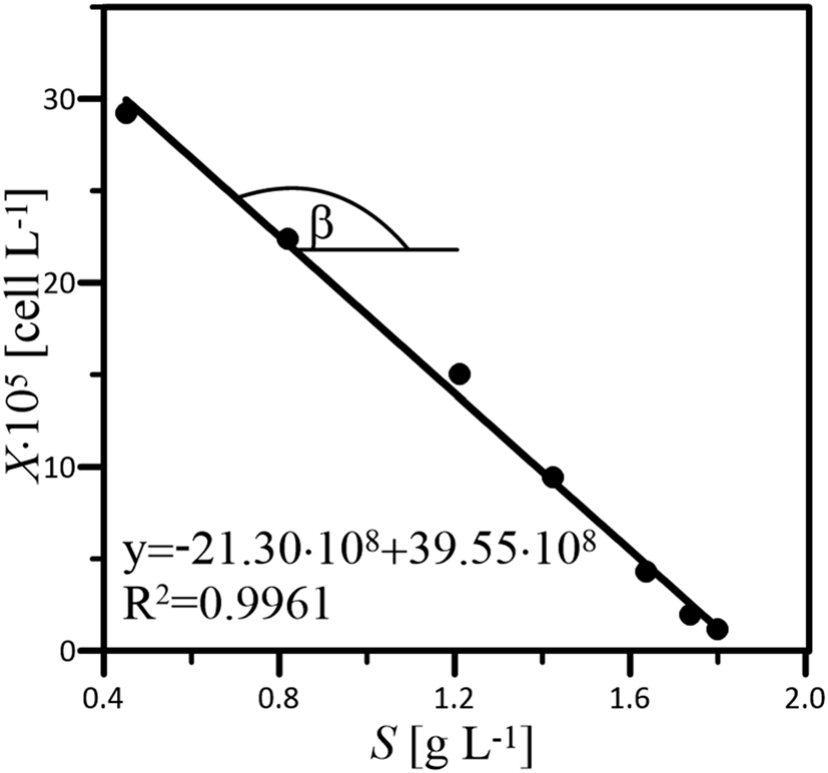
Exemplary determination of Y^*^_X/S_ value by the graphical method applied in the case of the culture of HL-60 cells performed in WAVE 25 operated at *α* = 6° and *ω* = 20 min^−1^

Values of Y^*^_X/S_ describing the yield of cell growth per mass unit (i.e., 1 g) of a substrate (i.e., glucose) have been determined by the graphical method (Fig. 2) according to the following equation:

8 $$Y_{X/S}^{*} = - tg\beta \; \left[ {{\text{cells g}}_{{{\text{glc}}}}^{{ - {1}}} } \right].$$

Based on the biomass balance equation:

9 $$\frac{{{\text{d}}X}}{{{\text{d}}t}} = \mu_{{\max}} X,$$

the values of apparent *μ*_max_, which characterize the cell growth rate in process conditions, have been finally calculated as the derivative of *X* after *t* divided by *X*, as follows:

10 $$\mu_{{\max}} = \frac{{{\text{d}}X}}{{{\text{d}}t}}X^{ - 1} \; \left[ {{\text{h}}^{{ - {1}}} } \right].$$

## Results

### Values of Re_L_ and ***k***_L_***a***

First of all, the values of Re_L_, as well as *k*_L_*a*, have been calculated for each set of operational parameters (i.e., *α* and *ω*) defining hydrodynamic conditions of wave-induced agitation during cultures of HL-60 cells performed in WAVE 25 bioreactor. The values of Re_L_ for the culture medium subjected to wave-induced mixing were calculated based on Eq. (6). In the case of values of *k*_L_*a* coefficient, the original dimensionless correlation for the prediction of *k*_L_*a*, which has been presented as Eq. (7) (i.e., equation previously introduced in [10]), might be significantly simplified, according to performing all experiments under unchanged *Q*_G_ equaled to 0.5 L min^−1^ (please see Table 1). Such approach allows to assume constant values of Re_G_ (i.e., Re_G_ = 6.293), as well Sc (i.e., Sc = 228.24), for all performed cultures, which have been all carried out in the same type of Cellbag characterized by *L* equaled to 0.32 m, with the culture medium incubated at 37 °C and characterized by *D*_L_ equaled to 3.061 × 10^–9^ m^2^ s^−1^ [20]. Finally, it resulted in the following simplified form of dimensionless correlation applied to predict the *k*_L_*a* coefficients in the WAVE 25 bioreactor operated at *Q*_G_ = 0.5 L min^−1^ with the liquid phase incubated at 37 °C:

11 $$k_{{\text{L}}} a = 8.57 \times 10^{ - 2} {\text{Re}}_{{\text{L}}}^{0.522} \; \left[ {{\text{h}}^{{ - {1}}} } \right].$$

The list of data summarizing operational parameters of WAVE 25 defining condition of wave-induced agitation (i.e., *α* and *ω*), and the correlated values of Re_L_ and *k*_L_*a* which were reached in the performed cultures of HL-60 cells, has been presented in Table 2.

**Table 2:** The values of Re_L_ and *k*_L_*a* reached for cultures of HL-60 cells performed in WAVE 25 at various values of *α* and *ω* as operational parameters defining wave-induced agitation

*α* (°)	*ω* (min^−1^)	Re_L_ (–)	*k*_L_*a* (h^−1^)
6	2	510	2.83
	20	5104	9.43
	40	10,208	13.55
2	20	1704	5.32
6		5104	9.43
12		10,152	13.51

Re_L_ and *k*_L_*a* might reach similar values for various pairs of *α* and *ω* defining conditions of wave-induced agitation in WAVE 25 bioreactor. In our experiments, the highest value of Re_L_ equaled to 10,208 was calculated for *α* and *ω* set as 6° and 40 min^−1^, respectively. But very similar value of Re_L_ equaled to 10,152 characterized the flow of waving liquid phase in Cellbag at *α* and *ω* equaled to 12° and 20 min^−1^, respectively. In other words, almost the same values of Re_L_ have been independently reached for two cultures performed for two sets of operational parameters which differed in doubled *α* and twice reduced *ω*. Thus, it is clearly seen that very similar values of Re_L_ can be obtained for two independent sets of *α*-and-*ω* pairs, which are differing significantly in values of operational parameters. Such effects probably resulted from differently applied values of paired operating parameters (i.e., *α* and *ω*) in cultures characterized by almost the same values of Re_L_ (i.e., maximal *α* and moderate *ω* for Re_L_ = 10,152 vs. moderate *α* and maximal *ω* for Re_L_ = 10,208). The lowest value of Re_L_ was estimated for oscillations defined by 6° and 2 min^−1^, and it was the only case of wavy flow of culture medium characterized by Re_L_ less than 1,000.

In the case of the *k*_L_*a* coefficient predicted for the studied system, the highest values exceeding level of 13 h^−1^ have been reached for the waving of culture medium at the highest *α*, as well as for the most intensive *ω*. Definitely, the lowest value of *k*_L_*a* characterized wave-induced agitation defined by *α* and *ω* equaled to 6° and 2 min^−1^, respectively, and in such case, *k*_L_*a* less than 3 h^−1^ could only be reached in the studied system.

### DO profiles

**Fig. 3:**
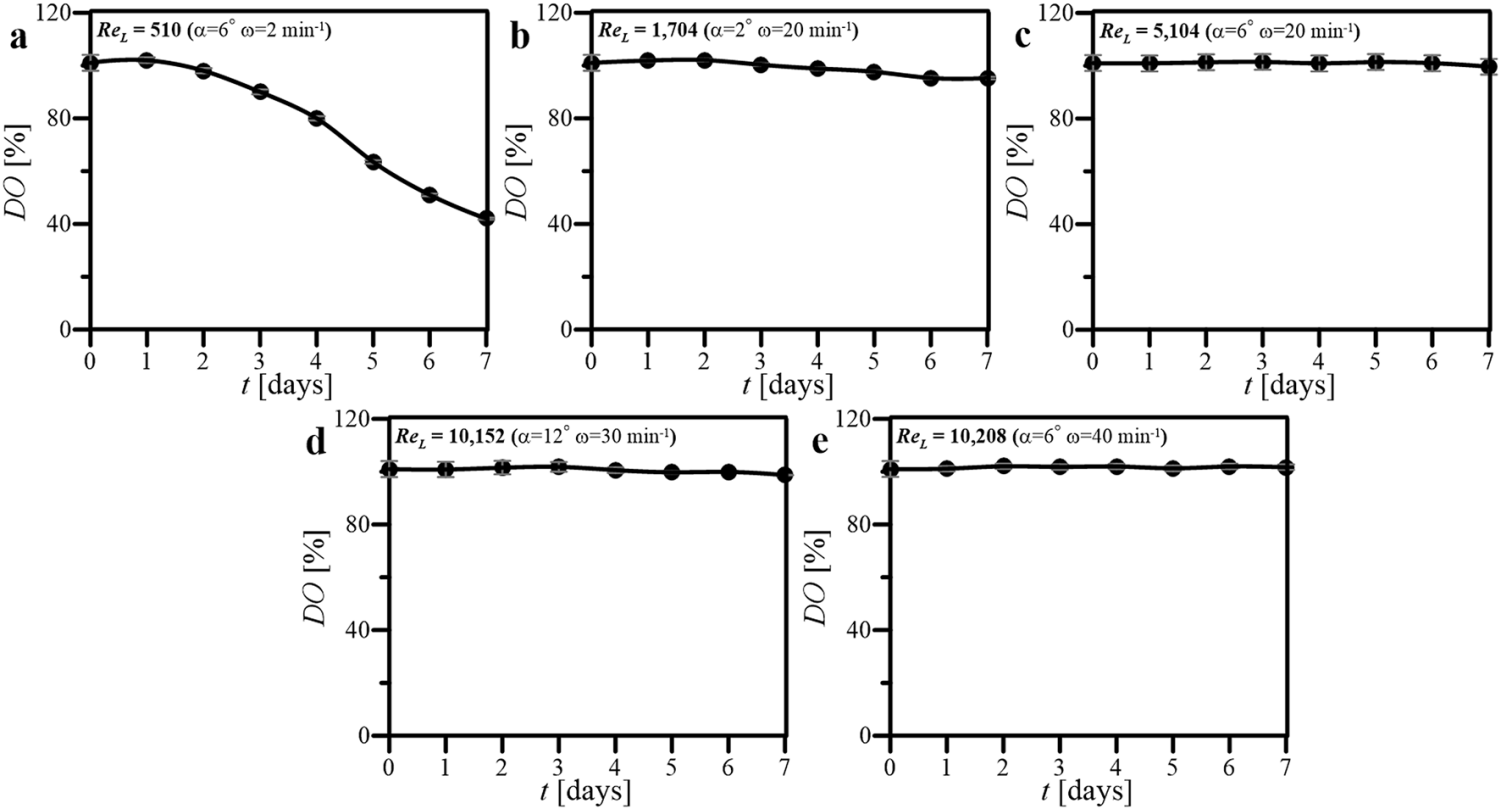
DO profiles obtained for cultures of HL-60 cells performed at various values of Re_L_ characterizing wavy flow of culture medium in oscillatory rocked WAVE 25 (**a**–**e**)

DO profiles characterizing culture conditions of all studied bioprocesses performed in WAVE 25 at defined wave-induced agitation have been presented in summarized form in Fig. 3.

Only one oscillatory rocked culture system, in which the flow of waving liquid phase has been characterized by the lowest value of Re_L_ equaled to 510 (i.e., for *α* = 6° and *ω* = 2 min^−1^), was not sufficiently aerated, which resulted in progressive decrease in DO level started on the 3rd day of culture (Fig. 3a). For the rest of cultures of HL-60 cells performed in WAVE 25 at Re_L_ varied from 1704 to 10,208 (Fig. 3b–e), the level of DO retained its maximal value, i.e., 100%, for whole period of 7 days. Such results definitely indicated that metabolism of the HL-60 cells, as well as their proliferation, would not be limited by the availability of O_2_ dissolved in culture medium.

### Density of HL-60 cells and their viability

**Fig. 4:**
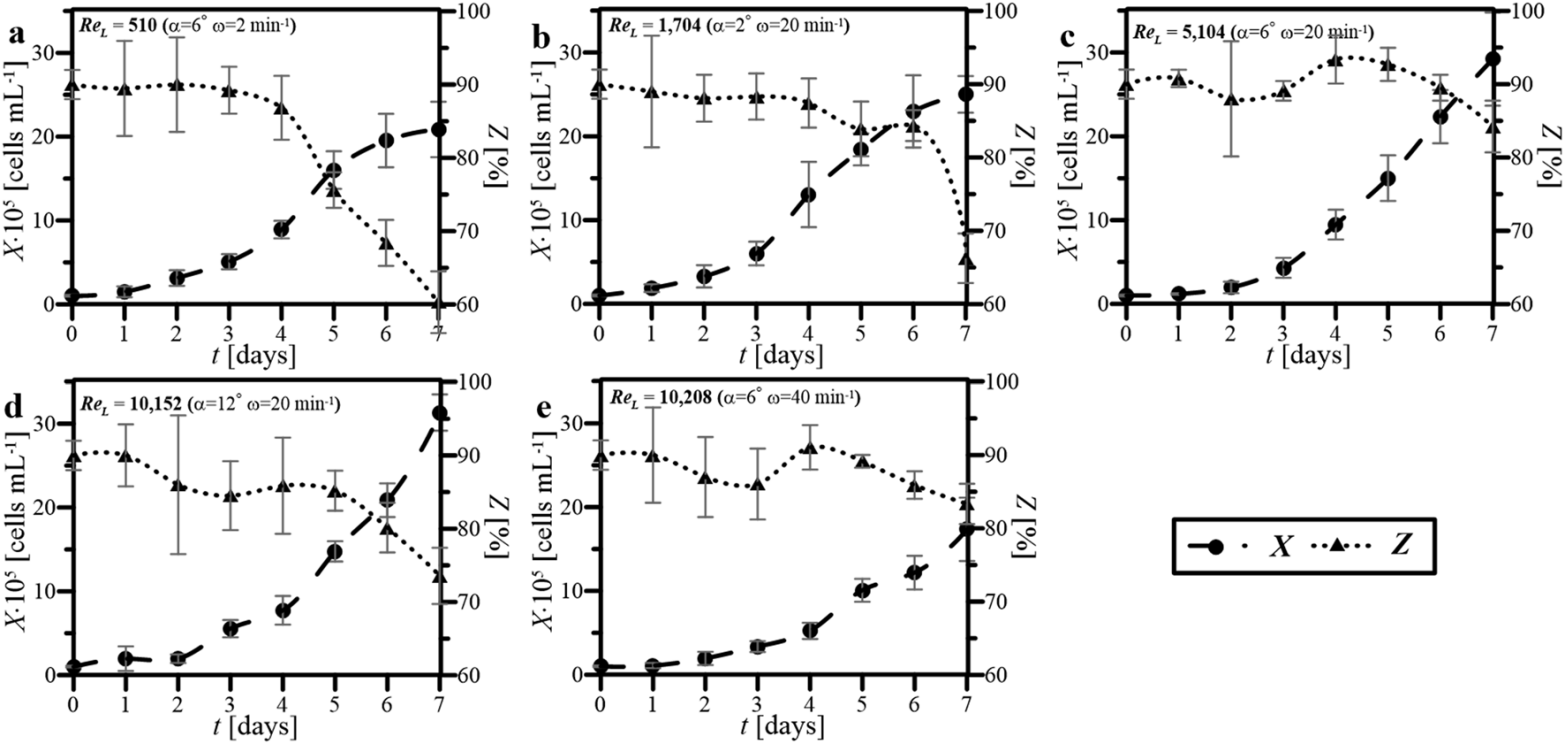
*X* and *Z* profiles obtained for cultures of HL-60 cells performed in WAVE 25 at various values of Re_L_ characterizing flow of waving culture medium (**a**–**e**)

The values of *X*, as well as *Z*, determined for HL-60 cells maintained in WAVE 25 under studied conditions of wave-induced agitation have been presented as related graphs in Fig. 4.

The highest value of *X* equaled to ca. 3 × 10^6^ cells mL^−1^ has been reached on 7th day for the cultures characterized by Re_L_ equaled to both 5104 (Fig. 4c) and 10,152 (Fig. 4d), which characterized cultures performed with the following two sets of operation parameters: *α* = 6°, *ω* = 20 min^−1^ and *α* = 12°, *ω* = 20 min^−1^, respectively. But cultures performed at such conditions of wave-induced agitation differed in maximal values of *Z* reached for them (Fig. 4c): 93% viability has been reached on the 4th–5th day of culture performed at Re_L_ = 5104 (it was also the highest *Z* obtained for all performed cultures) (Fig. 4c), and Z less than 85% obtained for bioprocess performed at Re_L_ = 10,152 (Fig. 4d). What was also interesting is that the significant influence of the value of *α* (at constant value of *ω*) on the level of reached *X* has been observed (please see Fig. 4b–d). The influence of the value of *ω* (at constant value of *α*) was rather minor (please see Fig. 4a, c, d). Furthermore, the influence of the value of *ω* (at constant value of *α*) on the level of obtained *Z* has been unequivocally much significant than in the case of cultures performed at various values of *α* but constant value of *ω*. In the case of culture performed at the lowest estimated value of Re_L_ equaled to 510 (Fig. 4a), the significant progressive decrease in the value of *Z* after the 4th day must also be noticed.

### Metabolic activity of HL-60 cells and glucose consumption

The activity of HL-60 cells was monitored daily based on two independently performed analyses: by the determination of *a*_m_ values, which quantitatively characterized activity of intracellular oxidoreductases, and by estimation of r^*^_glc/cell_ values, as parameter which characterized the average glucose consumption rate by single cell cultured in the studied systems.

**Fig. 5:**
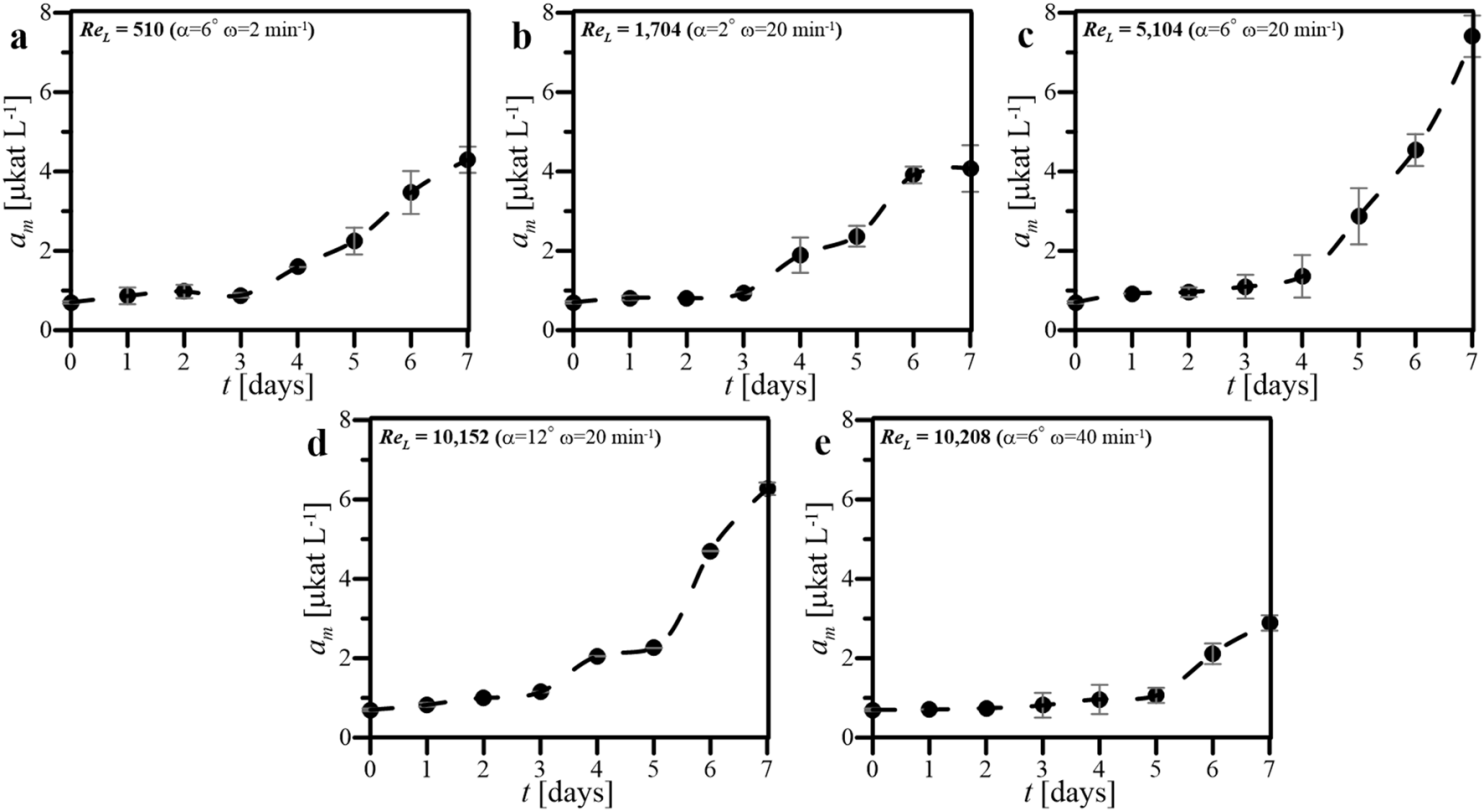
Values of *a*_m_ obtained for cultures performed in WAVE 25 at various hydrodynamic conditions of wave-induced agitation characterized by various Re_L_ (**a**–**e**)

The values of *a*_m_ determined for cultures of HL-60 cells maintained under various hydrodynamic conditions of wave-induced agitation in WAVE 25 have been presented in Fig. 5.

The significant increase in values of *a*_m_ have been observed after 3 days (Fig. 5a-e). The highest value of *a*_m_ (i.e., *a*_m_ = 7.4 μkat L^−1^) has been noted on 7th day of the culture with wave-induced agitation characterized by Re_L_ equaled to 5104 (Fig. 5c), i.e., at *α* = 6° and *ω* = 20 min^−1^. The comparably similar profiles of *a*_m_ changes have been noted for two of cultures performed at the constant *ω* equaled to 20 min^−1^, but which differed in values of *α* equaled to 6° and 12°, i.e., cultures characterized by Re_L_ equaled to 5104 (Fig. 5c) and 10,152 (Fig. 5d), respectively. Other cultures performed under condition of wave-induced agitation, i.e., for Re_L_ equaled to 510 (Fig. 5a), 1704 (Fig. 5b) as well as 10,208 (Fig. 5e), were characterized by markedly lower levels of *a*_m_ which were not higher than value of ca. 3–4 μkat L^−1^ on 7th day of these bioprocesses.

**Fig. 6:**
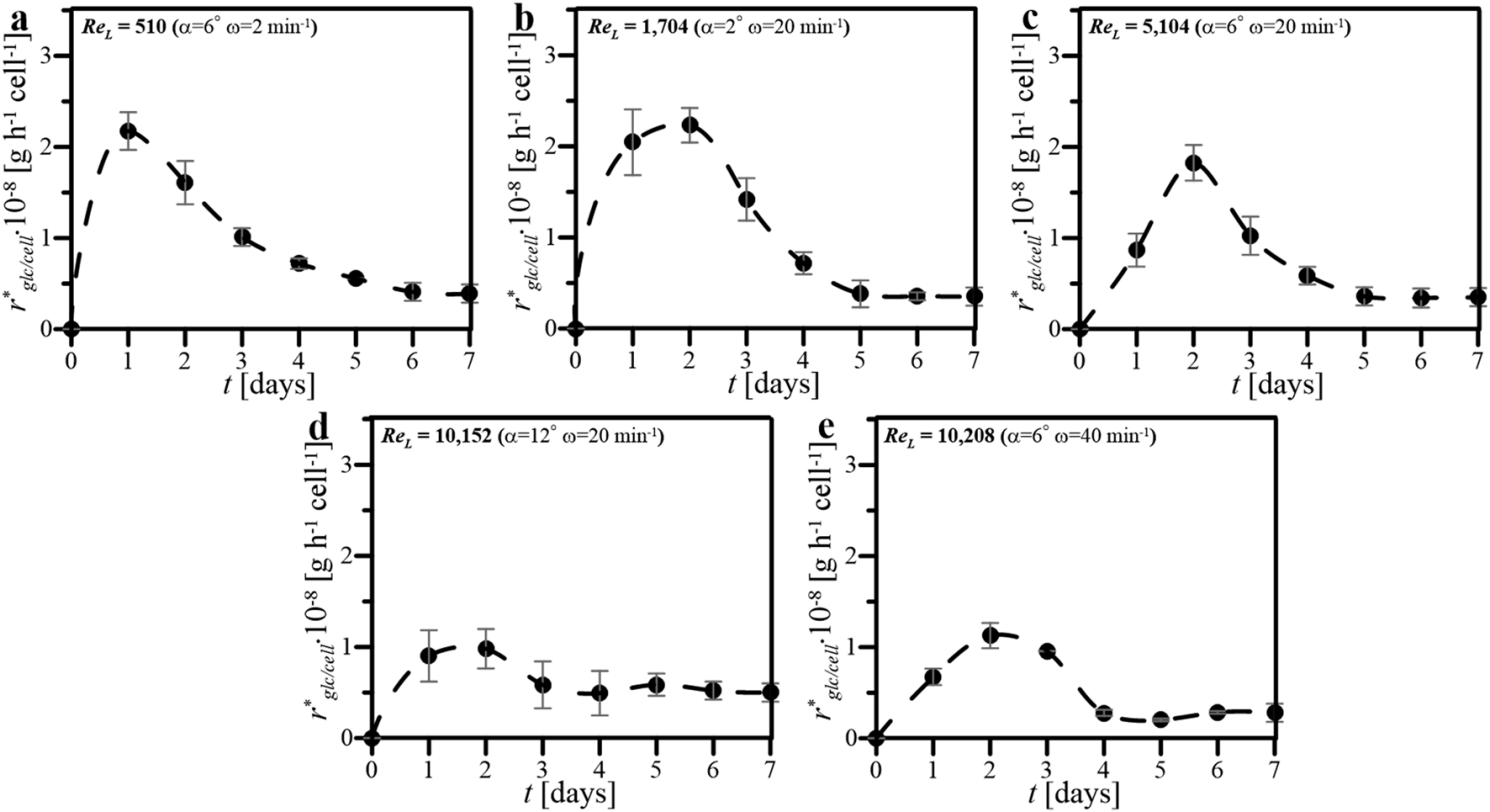
Values of r^*^_glc/cell_ estimated for and cultures performed in WAVE 25 at various hydrodynamic conditions of wave-induced agitation characterized by various Re_L_ (**a**–**e**)

The values of r^*^_glc/cell_ determined for cultures of HL-60 cells maintained in WAVE 25 under various conditions of wave-induced agitation bioreactor have been presented in Fig. 6.

In the case of 7-day cultures in WAVE 25, which were supported to wave-induced agitation, higher values of r^*^_glc/cell_ have been noted for first three days, than for later time-points of the cultures, regardless of waving conditions (Fig. 6a–e). The highest value of r^*^_glc/cell_ equaled to ca. 2.2 × 10^–8^ g h^−1^ cell^−1^ has been reached in 1st and 2nd days for two cultures performed at the lowest values of Re_L_, i.e., the cultures characterized by Re_L_ equaled to 510 (Fig. 4a) and 1,704 (Fig. 4b) and performed at the following two sets of operation parameters: *α* = 6°, *ω* = 2 min^−1^ and *α* = 2°, *ω* = 20 min^−1^, respectively. A common feature of all systems regardless of culture conditions was the stabilization of r^*^_glc/cell_ value at similar level, i.e., r^*^_glc/cell_ not higher than 0.5 × 10^–8^ g cell^−1^ h^−1^, for longer times of cultures, i.e., from 5th day up to the end of the experiment.

### Activity of LDH in culture medium

**Fig. 7:**
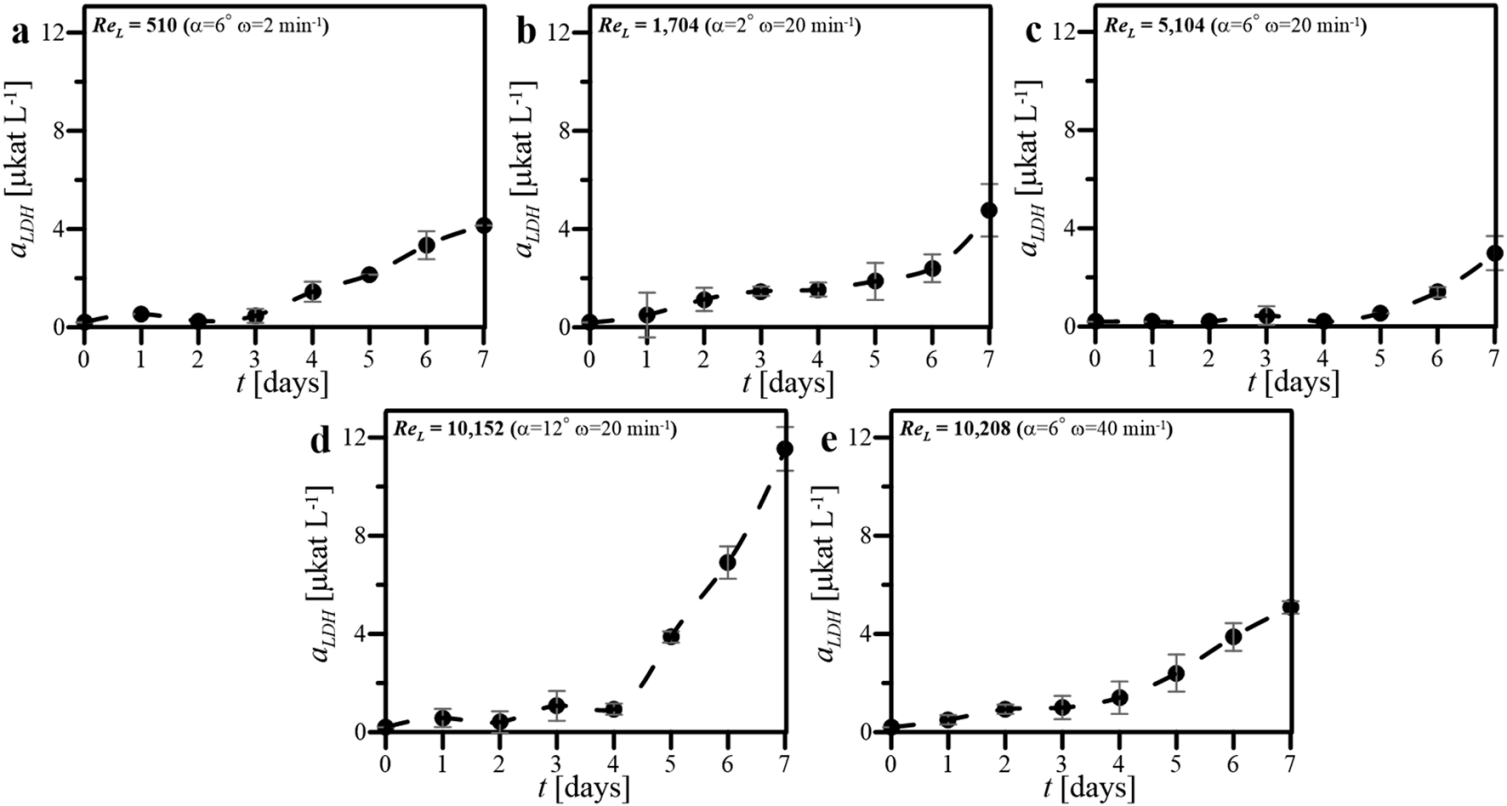
Values of *a*_LDH_ determined for cultures performed in WAVE 25 at various conditions of wave-induced agitation characterized by various Re_L_ (**a**–**e**)

The values of *a*_LDH_ determined colorimetrically in samples of culture media harvested from cultures of HL-60 cells maintained under various conditions of wave-induced agitation at various values of Re_L_ have been presented in Fig. 7.

In most cases of the cultures of HL-60 cells performed in WAVE 25 (Fig. 7a–c, e), except the culture characterized by Re_L_ = 10,152 (i.e., waving defined by *α* = 12° and *ω* = 20 min^−1^) (Fig. 7d), the profiles of *a*_LDH_ for the following time points of each experiments were analogous. Small, but noticeable difference from mentioned regularity was the very low level of *a*_LDH_ determined during first 5 days of the culture characterized by Re_L_ = 5104, i.e., *α* = 6° and *ω* = 20 min^−1^ (Fig. 7c), and clear break of the threshold of *a*_LDH_ detection value noted only in samples of the culture medium harvested at 6th and 7th days of this experiment. In the case of the culture rocked in WAVE 25 at the highest value of *α*, which was characterized by Re_L_ equaled to 10,152, i.e., the culture rocked at *α* = 12° and *ω* = 20 min^−1^ (Fig. 7d), the values of *a*_LDH_ determined in samples collected during last 3 days of this experiment were significantly higher (see Fig. 7a) if compared to *a*_LDH_ values noted for any other culture analyzed in our study. The highest value of *a*_LDH_ at 7th day of the experiment was equaled to 11.5 μkat L^−1^ (Fig. 7d), and it was over two times higher than the maximal value of *a*_LDH_ characterizing culture medium harvested from any other culture performed in our experiments.

## Discussion

Nonadherent hematopoietic cells are frequently recognized as ex vivo sources of protein-based bioproducts applied in biotechnology, and biomedicine as well. Up to now, HL-60 cells as valuable human cells, have been applied as model in many studies on blood cells differentiation and physiology [14–17], or as a simple in vitro model in determination of cytotoxicity of anti-leukemic drugs [18, 19]. But the efficiency of in vitro cultures of suspended biomass of HL-60 cells has not been extensively discussed so far, and the growth of HL-60 cells maintained in disposable bioreactor, as well as the influence of hydrodynamic conditions of the wave-type agitation on proliferation of HL-60 cells, has not been deeply studied or analysed up to date.

Figures 3, 4, 5, 6 and 7 comprehensively present the results of the cultures of human nonadherent HL-60 cells performed in WAVE 25 bioreactor under conditions of 2-D wave-assisted agitation characterized by various level of Re_L_. Any decrease of DO (Fig. 3a, b) resulted in drop of *Z* (Fig. 4a, b), which has been clearly noted for wave-type agitation characterized by Re_L_ 510 or 1704. The significant decrease of *Z* observed in Fig. 4a was probably connected with inefficient aeration which caused insufficient levels of DO observed under hydrodynamic conditions characterized by Re_L_ 510, i.e., the lowest studied level of Re_L_ in our experiments. In the case of metabolic activity of HL-60 cells, the clear influence of *X* level on *a*_m_ was observed: the increase of *X* resulted in increase of *a*_m_ (Fig. 3 vs. Figure 5). High values of *a*_m_ observed in cultures performed at Re_L_ 5104 (Fig. 5c), probably resulted from high values of *X* (Fig. 4c) reached under these hydrodynamic conditions. Rather low values of *a*_m_ which were noted for bioprocess performed at Re_L_ 10,208 (Fig. 5e), probably resulted from low values of *X* (Fig. 4e) reached in this culture. Also the levels of r^*^_glc/cell_ reached in the cultures were probably correlated with hydrodynamic conditions under which the culture was performed. The increase of Re_L_ resulted in the decrease of r^*^_glc/cell_ (Fig. 6). In the case of activity of LDH noted in culture media, the reached level of *a*_LDH_ was probably correlated with the values of *Z*: the decrease of *Z* resulted in the increase of *a*_LDH_ (Figs. 3 vs. 7). Rather low values of *a*_LDH_ which has been noted for bioprocess performed at Re_L_ 5104 (Fig. 7c), probably resulted from high values of *Z* (Fig. 4c) observed in such culture. The highest value of *a*_LDH_ has been identified in cultures characterized by Re_L_ 10,152 (Fig. 7d), and what corresponds with rather moderate level of *Z* (but noted at the highest level of *X*) (Fig. 4d). More LDH released from higher number of damaged cells resulted in higher levels of *a*_LDH_. Moreover, the bioprocess characterized by Re_L_ 10,152 was performed at conditions of wave-induced agitation at *α* = 12° and *ω* = 20 rpm, so at the highest setup of *α* supported by WAVE 25 bioreactor and at moderate level of *ω*. In the case of the bioprocess performed under hydrodynamic conditions characterized by similar value of Re_L_ 10,208, i.e., performed at *α* = 6°, *ω* = 40 rpm (so at the highest setup of *ω* supported by WAVE 25 bioreactor and at moderate level of *α*), destruction of biomass was not so intensive (Fig. 7e). Such results hypothetically prove that the destruction of HL60 cells was more intensive at higher values of *α* applied than at higher values of *ω*.

The results presented above in Figs. 3, 4, 5, 6 and 7 undoubtedly confirmed the correctness of applicability of the single-use bioreactor supported with wave-assisted agitation for efficient propagation of HL-60 cells. But the direct comparison of our data with any previously published results on bioprocess involving HL-60 cells and performed in disposable bioreactor system is not possible due to the lack of referenced literature data. However, the propagation of nonadherent HL-60 cells, as well as the results of in vitro performed cultures of such cells, can be compared and referred in a reasonable way to the results of bioprocesses involved other cells of mammalians, which were maintained in vitro in suspended form. The range of literature data on cultures of various types of nonadherent cells which were performed in two main commercially available systems of disposable bioreactors supporting wave-type agitation assisted by oscillatory 2-D rocking motion, i.e., Biostat^®^ RM equipped with CultiBag^®^ and *ReadyToProcess* WAVE™ equipped with CellBag^®^, which are manufactured by Sartorius and GE Healthcare, respectively, has been presented in Table 3.

**Table 3:** Previously published literature data on cultures of human, mammalian and insect cells performed in two main commercially available disposable bioreactors with 2-D wave-type agitation (data for the static, i.e., unagitated, culture are presented referentially)

Bioreactor system (manufacturer)	*V* (L)	Cell line (origin)	*X* (cells × mL^−1^)	*μ*_max_ (h^−1^)	Y^*^_X/S_ (10^8^ cells g_glc_^−1^)	References
Static conditions (as reference data)	0.05 (in 75-cm^2^ flasks)	HL-60 (human)	2.5 × 10^6^	0.023	13.86	[21]
Biostat^®^ RM equipped with Cultibag^®^ (by Sartorius, DE)	1.0 (in 2-L bag)	RBC (human)	4.9 × 10^5^	n/a	n/a	[22]
	5.0 (in 10-L bag)	CHO (hamster)	5.0 × 10^6^	n/a	n/a	[23]
*ReadyToProcess* WAVE™ equipped with Cellbag^®^ (by GE, US)	0.45 (in 2-L bag)	BHK21 (hamster)	1.8 × 10^6^	n/a	n/a	[24]
	1.0 (in 2-L bag)	HEK 293 (human)	2.7 × 10^6^	n/a	n/a	[25]
	1.0 (in 20-L bag)	NS0 (murine)	2.3 × 10^6^	n/a	n/a	[25]
	1.5 (in 20-L bag)	HEK 293 (human)	1.5 × 10^6^	n/a	n/a	[26]
	3.0 (in 20-L bag)	NS0 (murine)	2.1 × 10^6^	n/a	n/a	[25]
	10.0 (in 20-L bag)	NS0 (murine)	5.0 × 10^6^	n/a	n/a	[25]
	0.30 (in 2-L bag)	HL-60 (human)	3.1 × 10^6^	0.038	25.64	current study

In the case of two our cultures characterized by Re_L_ equaled to 5104 and 10,152, i.e., cultures of human leukemia HL-60 cells, which have been performed for two sets of parameters defining the wave-induced agitation by the same value of *ω* = 20 min^−1^ but various values of *α* (i.e., equaled to 6° and 12°, respectively), the most robust growth of HL-60 cells characterized by value of *X* equaled to ca. 3 × 10^6^ cells mL^−1^ in both cases, has been obtained. Thus, the maximal values of *X* observed in our experiments are consistent with range of previously published values of *X* for cultures of various types of nonadherent cells performed under conditions of wave-assisted agitation in various culture bags varying in total volume, as well as in volume of culture medium, which have been presented in Table 3. In the case of previously published results of human cells maintained in single-use bioreactor system, the highest value of *X*, i.e., 2.7 × 10^6^ cells mL^−1^, has been reported by Singh [25] and it characterized biomass of HEK 293 cells (i.e., human embryonic kidney cells) maintained in the RM bioreactor equipped with 2-L Cultibag filled with 1.0 L culture medium. Such value corresponds to the maximal value of *X* reached in our experiments (i.e., 3 × 10^6^ cells mL^−1^). In the case of other mammalian cells propagated in disposable bioreactor under wave-assisted agitation, the highest value of *X*, i.e., 5.0 × 10^6^ cells mL^−1^, has been reached in two independent cultures: Chinese hamster ovary epithelial CHO cells maintained in the RM bioreactor with 10-L Cultibag filled with 5.0 L culture medium, as reported by Eibl et al. [23], as well as murine myeloma NS0 cells maintained in the WAVE bioreactor equipped with 20-L Cultibag filled with 10.0 L culture medium, as once more reported by Singh [25].

The values of *X* and *Z,* which jointly characterized the increase and condition of HL-60 cells biomass maintained in the cultures performed in WAVE under conditions of wave-type agitation, were both generally rather consistent with values reached typically in static cultures of HL-60 cells performed at laboratory scale in standard unagitated conditions, which have been published previously [21]. Much significantly differences have been noted for other parameters which quantitatively characterized HL-60 cells maintained in the disposable wave-agitated system, i.e., *a*_m_, r^*^_glc/cell_ and *a*_LDH_. Definitely higher values of *a*_m_ have been noted in the case of all cultures supported by wave-assisted agitation. The static culture has been characterized by higher values of r^*^_glc/cell_ noted for following days of culture, if compared to level of r^*^_glc/cell_ reached at the same time points for waving cultures. Moreover, the values of maximal r^*^_glc/cell_ reached in the particular cultures have decreased with the increase of Re_L_. In the case of level of *a*_LDH_ detected in the cultures, the applying of wave-type agitation did not escalate the releasing of LDH from cells, what might be generally interpreted as the lack of significant negative influence of oscillatory performed wave-assisted agitations supported by WAVE 25 on the cell integrity of HL-60 cells maintained under such culture conditions. The several-fold increase in *a*_LDH_ value has been only seen for the culture characterized by Re_L_ equaled to 10,152, i.e., performed at *α* = 12° and *ω* = 20 min^−1^, what could be interpreted as higher influence of *α* than *ω*, on negative side effects of shear stress generated in the oscillatory operating culture bag.

The maximal value of *X* reached in our experimental cultures of HL-60 cells was in line with data on other human cells cultured in disposable culture bags mounted in 2-D oscillatory driven bioreactor systems. Nevertheless, the presentation of apparent *μ*_max_ and Y^*^_X/S_ values calculated based on Eqs. 8 and 10, respectively, characterizing proliferation of HL-60 cells in our experiments (Table 4), should give more precise information on efficiency of blood-derived nonadherent cells propagation performed in WAVE 25 operating at various parameters of wave-assisted agitation.

**Table 4:** Values of apparent *μ*_max_ and Y^*^_X/S_ characterizing the efficiency of HL-60 cells proliferation in cultures performed under various regimes of wave-assisted agitation characterized by various Re_L_

Re_L_ (–)	*µ*_max_ (h^−1^)	Y^*^_X/S_ (10^8^ cells g_glc_^−1^)
510	0.0241	25.64
1704	0.0361	15.81
5104	0.0380	21.30
10,152	0.0328	19.15
10,208	0.0305	14.81

The highest value of apparent *μ*_max_, i.e., *μ*_max_ = 0.0380 h^−1^, has been reached for the culture performed at Re_L_ equaled to 5104, i.e., at *α* = 6° and *ω* = 20 min^−1^, whereas the culture performed at Re_L_ equaled to 510, i.e., at *α* = 6° and *ω* = 2 min^−1^ resulted in value of Y^*^_X/S_ equaled to 25.64 × 10^8^ cells g_glc_^−1^, which was the highest value of Y^*^_X/S_ noted in our experiments. Thus, the highest values of apparent *μ*_max_ and Y^*^_X/S_ noted for the HL-60 cells maintained in WAVE 25 were 65% and up to 85%, respectively, higher than values of those parameters calculated for the static culture performed referentially without any agitation (view Table 3.).

Unfortunately, the data on values of apparent *μ*_max_ which have been obtained in the present study can only be limitedly compared to the previously presented literature data characterizing cultures of other types of nonadherent cells, which are presented in Table 3. The reason is the lack of quantitative data on the growth rate (e.g.,*μ*_max_ or apparent *μ*_max_) for human cells cultured in disposable bioreactors under conditions of wave-type agitation, available in the literature. To the best of our knowledge, the quantitative data on the growth rate of HL-60 cells, for their proliferation under conditions of wave-agitation, have not yet been published so far.

In the case of estimated values of Y^*^_X/S_, it can be clearly seen that the highest yield of growth of HL-60 cells per 1 g of consumed glucose (i.e., Y^*^_X/S_ = 25.64 × 10^8^ cells g_glc_^−1^) has been obtained for culture characterized by Re_L_ equaled to 510. Such culture system has been obtained when the culture bag was oscillatory rocked at moderate level of *α*, i.e., *α* equaled to 6°, and at the lowest preset of *ω*, i.e., *ω* equaled to 2 min^−1^, what finally resulted in the lowest value of Re_L_ characterized the flow of the culture medium inside the bag, among all cultures performed under conditions of wave-assisted agitation. Furthermore, we hypothesize that the culture conditions obtained in WAVE at Re_L_ equaled to 510 did not resulted in escalation of negative side-effects of hydrodynamic shear stress on fragile HL-60 cells maintained under such gentle culture conditions. Thus, at Re_L_ equaled to 510, HL-60 cells could probably retained their native morphology unchanged, as well as retain their metabolic pathways at level specific to typical conditions of the static culture without any kind of agitation applied, but under sufficient level of aeration, which has resulted in the highest value of Y^*^_X/S_.

Re_L_ numbers estimated for two independently performed cultures reached similar values (i.e., Re_L_ = 10,152 at *α* = 12° and *ω* = 20 min^−1^ and Re_L_ = 10,208 at *α* = 6° and *ω* = 40 min^−1^). In other words, almost the same values of Re_L_ have been independently reached for two cultures performed at two set of operational parameters which differed in doubled *α* and twice reduced *ω*. Thus, it is clearly seen that very similar value of Re_L_ can be obtained for two independent sets of *α*-and-*ω* pairs, which differ significantly in values of operational parameters. Despite similar Re_L_ values, the differences in the values of *X*, *a*_m_ and *a*_LDH_ has been clearly observed for such cultures characterized by almost the same values of Re_L_ (Figs. 4d–e, 5d–e, 7d–e). We hypothesize that, instead of similar hydrodynamics of medium flow inside culture bag in the case of cultures characterized by very similar Re_L_, the levels of shear stress effects, which significantly influenced on HL-60 cells, were not the same. Thereby, the level of negative effects influencing on suspended cells could hypothetically significantly varied according to potential cell-destroying effects of hydrodynamical shear stress generated variously in two compared cultures characterized by similar values of Re_L_ but differing in values of *α* and *ω* at which the culture bag was oscillatory rocked.

## Conclusions

The results of the cultures of human nonadherent HL-60 cells performed under conditions of 2-D wave-assisted agitation supported by WAVE 25 bioreactor at various Re_L_ number have been comprehensively compared in details. The results of our experiments confirmed the feasibility and application benefits of bioreactor system supporting wave-type agitation provided by oscillatory 2-D rocking motion in high cell density cultures of nonadherent cells. This has been unequivocally proved by values of the *k*_L_*a* coefficient, i.e., from the range 2.83–13.55 h^−1^, estimated based on simplified dimensionless correlation for various presets of WAVE 25 system. Additionally, the values of Re_L_ for the flow of culture medium subjected to wave-induced mixing were calculated, and its values varied from 510 to 10,208, for the hydrodynamic conditions defined by studied extreme presets of WAVE 25, i.e., *α* = 6° at *ω* = 2 min^−1^ and *α* = 6° at *ω* = 40 min^−1^, respectively.

In the case of the culture aimed to obtain the highest growth rate, the different conditions of 2-D wave-assisted agitation should be applied than for the culture aimed to obtain the highest yield of cell growth per mass unit: the maximal values of apparent *μ*_max_ (i.e., *μ*_max_ = 0.038 h^−1^) and Y^*^_X/S_ (i.e., Y^*^_X/S_ = 25.64 × 10^8^ cells g_glc_^−1^) have been reached at Re_L_ equaled to 5,104 and 510, respectively. We strongly believe that the investigations performed in our studies are cognitively justified and they will simplify the scale-up of bioprocesses with nonadherent cells, and in particular human hematopoietic cells, utilizing the disposable bioreactors presenting similitude, and other disposable systems or spinner flasks.

## List of symbols

*A*_W_: Specific absorbency, –
*a*_LDH_: Activity of lactate dehydrogenase, µkat L^−^^1^
*a*_m_: Metabolic activity, µkat L^−^^1^
*C*_CO2_: CO_2_ concentration in the gas phase, %
*C*_O2_: O_2_ concentration in the gas phase, %
*D*_*L*_: O_2_ diffusion coefficient in water, m^2^ s^−^^1^
*d*: Dilution factor, –
*k*: Number of hemocytometer squares occupied by cells, –
*k*_L_*a*: Volumetric liquid-side mass transfer coefficient, h^−^^1^
*L*: Length of culture bag, m
Re_L_: Reynolds number for liquid phase, –
Re_G_: Reynolds number for gas phase, –
*r*^***^_glc/cell_: Specific glucose consumption rate, g h^−^^1^ cell^−^^1^
Sc: Schmidt number for liquid phase, –
*T*: Temperature, °C
*Q*_G_: Gas flow rate, L min^−^^1^
*V*_L_: Volume of culture medium, L
*X*: Density of HL-60 cells in culture medium, cell mL^−^^1^
*X*_0_: Initial density of HL-60 cells at starting point of culture, cell mL^−^^1^
*x*: Number of cells counted in hemocytometer, –
*Y*^***^_*X/S*_: Specific yield of biomass from subtract, cells g_glc_^−^^1^
*Z*: Viability of the cells, %
*z*: Number of alive cells,

## Greek symbols

*α*: Angle of oscillations, °
Δ*A*: Absorbency change, –
Δ*C*_glc_: Change of glucose concentration, g L^−^^1^
Δ*t*: Time interval, h
*µ*_max_: Apparent maximal specific growth rate, s^−^^1^
*ν*_L_: Liquid phase kinematic viscosity, m^2^ s^−^^1^
*ω*: Frequency of oscillations, min^−^^1^
*φ*: Average filter pore diameter, µm

## Subscripts

CO_2_: Carbon dioxide
O_2_: Oxygen

## Abbreviations

2-D: Two dimensional
ATCC: American Type Culture Collection
BHK21: Baby hamster kidney cell line
CBCU: Central bioreactor control unit
CHO: Chinese hamster ovary cell line
DO: Dissolved oxygen
FBS: Fetal bovine serum
HEK 293: Human embryonic kidney cell line
HL-60: Human leukemia white blood cell line
LDH: Lactate dehydrogenase enzyme
NS0: Nonsecreting murine myeloma cell line
PenStrep: Antibiotic mixture (of penicillin and streptomycin)
RBC: Red blood cell
RPMI: Roswell Park Memorial Institute 1640 medium
WAVE 25: *ReadyToProcess* WAVE™25 bioreactor
